# Antimicrobials cetylpyridinium-chloride and miramistin demonstrate non-inferiority and no “protein-error” compared to established wound care antiseptics *in vitro*

**DOI:** 10.3934/microbiol.2022026

**Published:** 2022-10-24

**Authors:** Julian-Dario Rembe, Vivian-Denise Thompson, Ewa Klara Stuermer

**Affiliations:** 1 Department of Vascular and Endovascular Surgery, Medical Faculty and University Hospital Duesseldorf, Heinrich-Heine-University, Duesseldorf, North-Rhine Westphalia, Germany; 2 Chair for Translational Wound Research, Center for Biomedical Education and Research, Witten/Herdecke University, Witten, North-Rhine Westphalia, Germany; 3 Department of Vascular Medicine, University Heart Center, University Medical Center Hamburg-Eppendorf (UKE), Hamburg, Germany

**Keywords:** cetylpyridinium chloride, miramistin, antimicrobials, antiseptics, wound infection

## Abstract

Concern about microbial tolerance and resistance to established antimicrobials drives research into alternatives for local antiseptic wound treatment. Precise efficacy profiles are thereby important in the evaluation of potential alternative antimicrobials, and protein interference (“protein error”) is a key factor.

Here, the antimicrobial efficacy of cetylpyridinium chloride (CPC) and miramistin (MST) was compared to the established antimicrobials octenidine (OCT), povidon-iodine (PVP-I), polyhexamethylene-biguanide (PHMB) and chlorhexidine (CHX). Efficacy was evaluated after 0.5, 1, 3, 5 and 10 min against *Staphylococcus aureus*, *Pseudomonas aeruginosa, Escherichia coli*, *Enterococcus faecium* and *Candida albicans* using an in vitro quantitative suspension method (based on DIN EN 13727). To investigate protein interference, 0.3% or 3% bovine albumin was used as the challenge.

OCT and PVP-I demonstrated a significant efficacy within 0.5 min, regardless of the microbial organism and protein challenge (*p* < 0.01). CPC and MST showed no inferiority in efficacy, with only MST needing up to 3 min to achieve the same microbial reduction. PHMB and CHX also achieved significant reduction rates over the tested time-course, yet demonstrated a necessity for prolonged exposure (up to 10 min) for comparable reduction. A protein interference was predominantly observed for PHMB against *S. aureus*, but without statistically significant differences in antimicrobial efficacy between the 0.3% and 3% protein challenges. All other tested agents showed no relevant interference with the presence of protein.

CPC and MST proved to be non-inferior to established wound antiseptics agents in vitro. In fact, CPC showed a more efficient reduction than PHMB and CHX despite there being an introduced protein challenge. Both agents demonstrated no significant “protein error” under challenging conditions (3% albumin), posing them as valid potential candidates for alternative antimicrobials in wound management.

## 1. Introduction

Wound infection remains a major challenge for healthcare providers and patients. An increasingly elderly patient population is at higher risk of developing surgical site infections (SSIs) and chronic wounds due to predisposing factors such as diabetes, peripheral arterial disease and chronic venous insufficiency [1]. Chronic wounds are generally considered to be colonized by microorganisms, and recent studies revealed up to 78% to have a biofilm [2]. SSIs account for, on average, 20% of hospital-acquired infections in Europe [3]–[6]. Antimicrobial wound cleansing and local antisepsis are key factors in the treatment of acute and chronic wound infections. Octenidin dihydrochloride/phenoxyethanol (OCT/PE), povidone iodine (PVP-I), polyhexamethylene biguanide hydrochloride (PHMB) and chlorhexidine (CHX) represent the main local antimicrobials used in wound management. So far, no single antimicrobial has proven to be generally superior in the treatment of local wound infection or promotion of wound healing [7]–[9]. With adverse effects on essential cells for skin regeneration, such as fibroblasts and keratinocytes, being reported for each agent [10],[11], antibiotic resistance on the rise [12], and first reports of developing tolerance to local antiseptic agents [13],[14], investigations into alternative antimicrobial agents with comparable robust efficacy and low cytotoxic impact to human skin cells are necessary.

Two potential alternatives are the quaternary ammonium compounds cetylpyridinium chloride (CPC) and miramistin (MST), belonging to the family of cationic surface-active agents. Based on the first promising results regarding antimicrobial efficacy and biocompatibility to human cells in vitro [15], this study was aimed to compare MST and CPC to OCT, PVP-I, PHMB and CHX in a side-by-side comparison. Since the local wound microenvironment represents a complex and challenging environment for any therapeutic agent with the potential for unwanted interaction, this aspect needs to be considered in a concise agent evaluation. Although it is not the only influential factor, the high level of protein content within a wound has proven to significantly impact an agent's efficacy in previous studies [16]–[18]; it has been demonstrated to the extent of complete efficacy loss under protein interference. This form of reduction in efficacy due to interference with proteins has been referred to as “protein error” in the field of wound antiseptics [16]. To account for this influence here, agents were evaluated in terms of their interference with protein as a proxy for potential performance loss in a wound environment. Short exposure times (as in clinical practice) against common wound pathogens (*Staphylococcus aureus*, *Escherichia coli*, *Pseudomonas aeruginosa*, *Enterococcus faecium* and *Candida albicans*) were chosen to obtain informative and clinically relevant data.

## 2. Materials and methods

### 2.1. Preparation of antiseptic solutions

OCT/PE (Octenisept®; 0.1%; Schülke & Mayr GmbH, Norderstedt, Germany) and PVP-I (Betaisodona®; 10%; Mundipharma GmbH, Limburg, Germany) were used as readily available customary products of everyday clinical use (concentrations as indicated by manufacturer). PHMB (20%; Bonding, Shanghai, China) and CHX (2%; Carl Roth, Karlsruhe, Germany) were prepared in sterile distilled water to reach the desired final test concentrations of 0.02% (v/v; PHMB) and 0.2% (v/v; CHX), respectively, in compliance with previously published studies.

Cetylpyridinium-chloride (CPC; Sigma-Aldrich, Schnelldorf, Germany) and Miramistin (MST; Farmhim, Shostka, Ukraine) were purchased as powders and also prepared in distilled water to reach comparative final test concentrations of 0.5% (w/v; CPC) and 0.05% (w/v; MST) based on previously published experiments [15]: such concentrations demonstrated a high antimicrobial efficacy without protein load, while at the same time exerting an acceptable biocompatibility compared to established agents. An overview as well as specifications for the tested products/substances is provided in table 1.

**Table 1.** Specifications on test substances. Information has been obtained from the manufacturer (commercially available products were used in undiluted formulations, as provided by the manufacturers).

Antimicrobial agent (commercial product)	Chemical group	Manufacturer	Composition	Final test concentration
Octenidin-dihydrochloride/phenoxy-ethanol (Octenisept®)	Bis-pyridinamine	Schülke & Mayr GmbH, Norderstedt, Germany	0.1% octenidin-dihydrochloride with 2% phenoxyethanol in 100mL aqueous solution	0.1 %
Polyhexamethylene-biguanide hydrochloride	Polymeric biguanide	Bonding, Shanghai, China	20% polyhexamethylene-biguanide hydrochloride stock solution in distilled water	0.02 %
Chlorhexidine gluconate solution 2%	Bis-biguanide	Carl Roth, Karlsruhe, Germany	2% chlorhexidine gluconate stock solution in distilled water	0.2 %
Povidone-iodine (Betaisodona®)	Iodine	Mundipharma GmbH, Limburg, Germany	10% povidone-iodine, glycerol, nonoxinol 9, disodium hydrogen phosphate, citric acid, sodium hydroxide, potassium iodate in aqueous solution	10 %
Miramistin (Miramistin®)	Quaternary ammonium compound (QAC)	Farmhim, Shostka, Ukraine	0.5 mg mL^−1^ miramistin powder in distilled water	0.05 %
Cetylpyridinium-chloride	Quaternary ammonium compound (QAC)	Sigma-Aldrich, Schnelldorf, Germany	5 mg mL^−1^ cetylpyridinium chloride in distilled water	0.5 %

### 2.2. Test organisms and nutrient solutions

In this work, *P. aeruginosa* (DSM-939), *S. aureus* (DSM-799), *E. coli* (DSM-11250), *E. faecium* (DSM-2146) and *C. albicans* (DSM-1386; all DSMZ, Braunschweig, Germany) were used as bacterial test strains. As the nutrient solution, sterile casein/soy peptone broth (CSB) was prepared, consisting of 15 mg mL^−1^ casein peptone, 5 mg mL^−1^ soy peptone and 5 mg mL^−1^ sodium chloride diluted in distilled water. The pH value was adjusted to 7.2 using 5N sodium hydroxide (all AppliChem, Darmstadt, Germany). One fresh colony of each bacterial strain was added to 50 mL of CSB and adjusted to initial counts of ~ 1.5–3.0 × 10^8^ CFU mL^−1^ (0.5 McFarland Standard) using a spectrophotometer. Fungal test solutions were prepared in the same manner by using malt/soy peptone broth and malt agar (MEA). Initial microbial CFU mL^−1^ counts were controlled by spreading serial dilutions of untreated microbial test solutions of each experiment onto agar plates, allowing exact calculations of reduction rates.

### 2.3. Challenge and neutralization solutions

To simulate a protein-rich wound environment and determine possible decreases in the efficacy of the tested antiseptics under high-protein conditions (protein interference), bovine albumin (Carl Roth, Karlsruhe, Germany) was added to the experimental setup as the challenge substance (as recommended in the standard DIN EN 13727 by the German institute for standardization (DIN)) [19]. Challenge solutions contained either 3 mg mL^−1^ (0.3%) or 30 mg mL^−1^ (3%) of albumin and were prepared using an autoclaved casein/natriumchloride solution dissolved in distilled water. The challenge solution was subsequently aliquoted and stored at –18 °C until usage.

To neutralize the antimicrobial activity of the active substances at the sampling time points, a neutralization solution was used based on recommendations in the DIN EN 13727; its neutralizing effect was validated for all tested antimicrobial agents; it was also verified as being non-bactericidal prior to experiments (data not shown). The neutralization solution comprised of 3 g L^−1^ of sodium thiosulfate, 30 g L^−1^ of saponine, 30 g L^−1^ of polysorbate 80 (Tween 80), 3 g L^−1^ of lecithin, 1 g L^−1^ of L-histidine and 1 g L^−1^ of L-cysteine (all Carl Roth, Karlsruhe, Germany), which were diluted in distilled water and autoclaved for sterility.

### 2.4. Quantitative suspension method

A modified quantitative suspension test method was used for the evaluation of antimicrobial efficacy; it was slightly adjusted to fit the purpose (methodology is based on DIN EN 13727) [19]. Briefly, 1 mL of microbial and 1 mL of challenge solution (0.3% or 3% bovine albumin) were carefully mixed for 2 min and 8 mL of antimicrobial test solution was added. After 0.5, 1, 3, 5 and 10 min of exposure, 1 mL of the resulting solution was transferred into the prepared neutralization solution (8 mL of neutralizer and 1 mL of distilled water) for 10 s of neutralization. Subsequently, the sample was serially ten-fold diluted, and 25 µL of each dilution step was plated on either CSA or MEA plates. The plates were incubated at 37 °C under aerobic conditions overnight; surviving microorganisms (in CFU mL^−1^) counted on the following day using a colony counter pen. For direct comparison of reduction rates against initial microbial counts, an untreated control of the initial bacterial test suspension was plated onto agar plates and evaluated in the same manner. Experiments were performed in triplicates at different times, and with two technical replicates each time (resulting in a total n = 6) for each tested antiseptic and microorganism challenged with either 3 mg mL^−1^ or 30 mg mL^−1^ of albumin solution.

### 2.5. Statistical analysis

Reduction rates were calculated for all tested antimicrobials (in Δlog_10_ CFU mL^−1^). For bacteria, a high antimicrobial efficacy (reducing at least 99.999% of initial bacterial counts) is indicated by a reduction of at least 5 log_10_ reduction steps, as specified in DIN EN 13727 [19]. For yeast, the cut-off for a high efficacy is considered to be at least 4 log_10_, as specified in DIN EN 13624. The mean values and standard error of the mean (SEM) were calculated and the differences considered statistically significant at *p* < 0.05.

Statistical evaluations of the antimicrobial efficacy were performed using a repeated measures two-way ANOVA with Geisser-Greenhouse correction. Tukey's test was used as post-hoc analysis for multiple comparisons in terms of comparative analyses between antiseptic agents. In terms of reduction over the exposure time-course, Dunnett's post-hoc analysis for multiple comparison was used. The statistics package GraphPad PRISM (GraphPad Software, Inc., La Jolla, United States of America) was used for statistical analysis.

## 3. Results

### 3.1. Antimicrobial efficacy on tested pathogens

An overview on the reduction rates (in Δlog_10_ CFU mL^−1^ ± SEM reduction steps compared to initial CFU mL^−1^) of the tested antimicrobial agents is given in Supplementary table 1.

#### 3.1.1. OCT/PE and PVP-I

**Figure 1.**
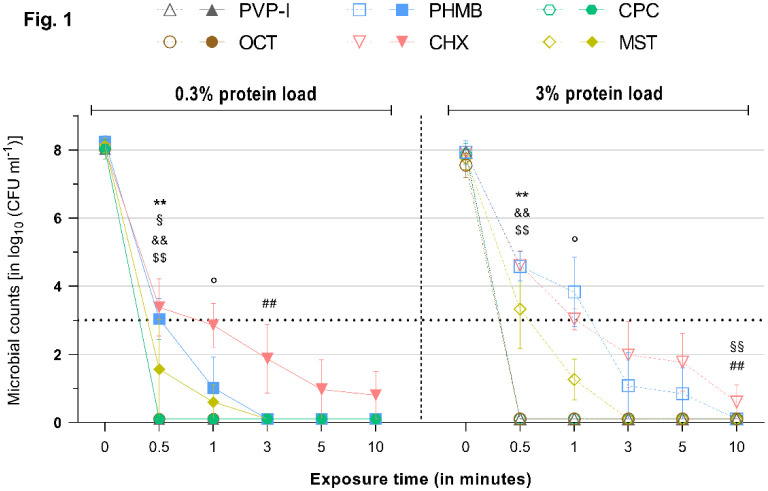
Reduction rates of tested antimicrobials against *S. aureus* under a 0.3% (left) or 3% (right) protein load. Microbial counts are expressed as log_10_ CFU mL^−1^ over time. Antimicrobials are displayed individually with a side-by-side comparison of the reduction rates under a 0.3% (left side–continuous lines and filled symbols) or 3% (right side–dashed lines and hollow symbols) protein load. Dotted horizontal line indicates the threshold set by the standards (DIN EN 13727 and 13624), i.e., a minimum required ≥5 log_10_ reduction steps for bacteria relative to the initial microbial counts, to achieve a reduction rate deemed “high”. Statistically significant reductions are marked at the first time point that significance was reached relative to the initial microbial counts, as indicated by different symbols for each tested antimicrobial (‘*’ ≙ CPC, ‘°’ ≙ MST, ‘§’ ≙ CHX, ‘#’ ≙ PHMB, ‘&’ ≙ PVP-I, ‘$’ ≙ OCT/PE). Degree of significance is indicated with the number of symbols (one–*p* < 0.05; two–*p* < 0.01; three–*p* <0.001, four–*p* < 0.0001).

**Figure 2.**
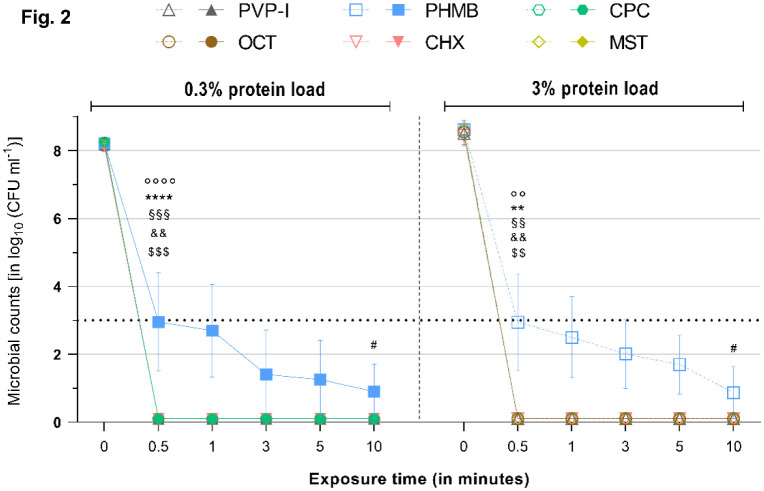
Reduction rates of tested antimicrobials against *E. coli* under a 0.3% (left) or 3% (right) protein load. Microbial counts are expressed as log_10_ CFU mL^−1^ over time. Antimicrobials are displayed individually with a side-by-side comparison of the reduction rates under a 0.3% (left side–continuous lines and filled symbols) or 3% (right side–dashed lines and hollow symbols) protein load. Dotted horizontal line indicates the threshold set by the standards (DIN EN 13727 and 13624), i.e., minimum required ≥5 log_10_ reduction steps for bacteria relative to the initial microbial counts, to achieve a reduction rate deemed “high”. Statistically significant reductions are marked at the first time point that significance was reached relative to the initial microbial counts, as indicated by different symbols for each tested antimicrobial (‘*’ ≙ CPC, ‘°’ ≙ MST, ‘§’ ≙ CHX, ‘#’ ≙ PHMB, ‘&’ ≙ PVP-I, ‘$’ ≙ OCT/PE). Degree of significance is indicated with the number of symbols (one–*p* < 0.05; two–*p* < 0.01; three–*p* <0.001, four–*p* < 0.0001).

**Figure 3.**
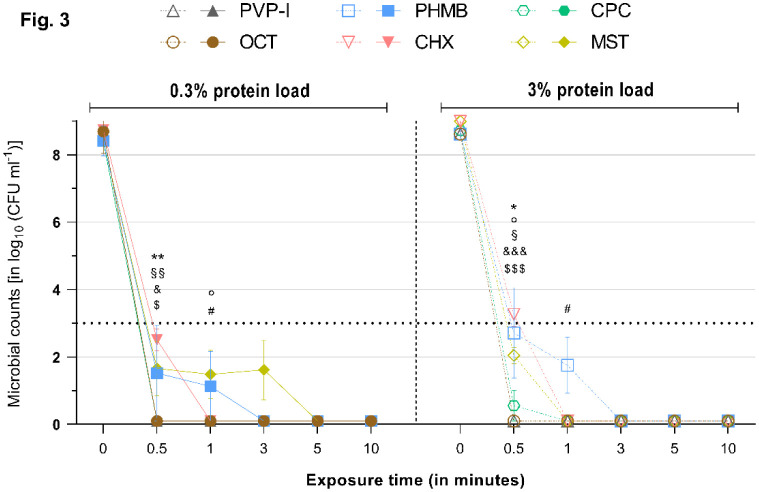
Reduction rates of tested antimicrobials against *P. aeruginosa* under a 0.3% (left) or 3% (right) protein load. Microbial counts are expressed as log_10_ CFU mL^−1^ over time. Antimicrobials are displayed individually with a side-by-side comparison of the reduction rates under a 0.3% (left side–continuous lines and filled symbols) or 3% (right side–dashed lines and hollow symbols) protein load. Dotted horizontal line indicates the threshold set by the standards (DIN EN 13727 and 13624), i.e., minimum required ≥ 5 log_10_ reduction steps for bacteria relative to the initial microbial counts, to achieve a reduction rate deemed “high”. Statistically significant reductions are marked at the first time point that significance was reached relative to the initial microbial counts, as indicated by different symbols for each tested antimicrobial (‘*’ ≙ CPC, ‘°’ ≙ MST, ‘§’ ≙ CHX, ‘#’ ≙ PHMB, ‘&’ ≙ PVP-I, ‘$’ ≙ OCT/PE). Degree of significance is indicated with the number of symbols (one–*p* < 0.05; two–*p* < 0.01; three–*p* <0.001, four–*p* < 0.0001).

**Figure 4.**
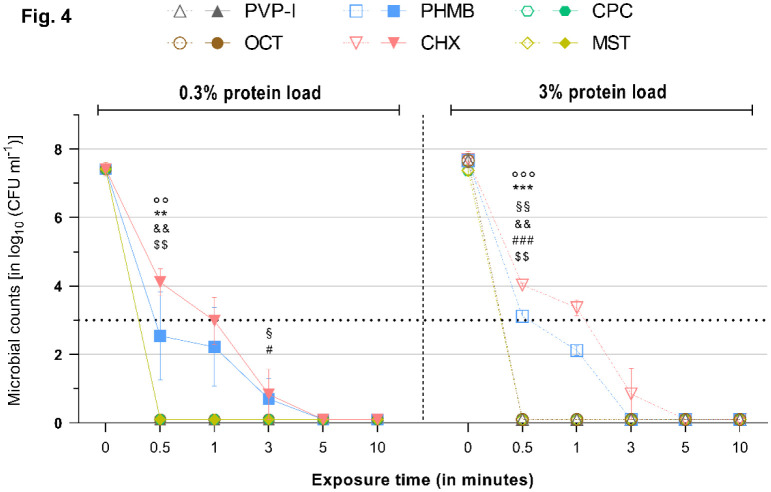
Reduction rates of tested antimicrobials against *E. faecium* under a 0.3% (left) or 3% (right) protein load. Microbial counts are expressed as log_10_ CFU mL^−1^ over time. Antimicrobials are displayed individually with a side-by-side comparison of the reduction rates under the 0.3% (left side–continuous lines and filled symbols) or 3% (right side–dashed lines and hollow symbols) protein load. Dotted horizontal line indicates the threshold set by the standards (DIN EN 13727 and 13624), i.e., minimum required ≥ 5 log_10_ reduction steps for bacteria relative to the initial microbial counts, to achieve a reduction rate deemed “high”. Statistically significant reductions are marked at the first time point that significance was reached relative to the initial microbial counts, as indicated by different symbols for each tested antimicrobial (‘*’ ≙ CPC, ‘°’ ≙ MST, ‘§’ ≙ CHX, ‘#’ ≙ PHMB, ‘&’ ≙ PVP-I, ‘$’ ≙ OCT/PE). Degree of significance is indicated with the number of symbols (one–*p* < 0.05; two–*p* < 0.01; three–*p* <0.001, four–*p* < 0.0001).

**Figure 5.**
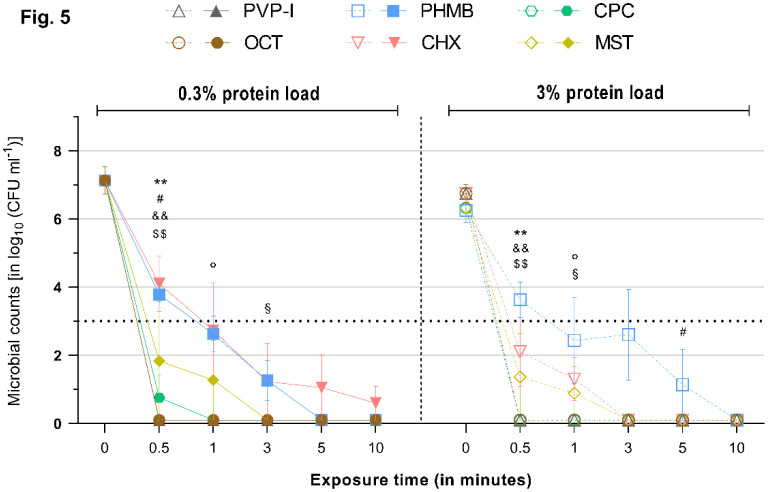
Reduction rates of tested antimicrobials against *C. albicans* under a 0.3% (left) or 3% (right) protein load. Microbial counts are expressed as log_10_ CFU mL^−1^ over time. Antimicrobials are displayed individually with a side-by-side comparison of the reduction rates under the 0.3% (left side–continuous lines and filled symbols) or 3% (right side–dashed lines and hollow symbols) protein load. Dotted horizontal line indicates the threshold set by the standards (DIN EN 13727 and 13624), i.e., minimum required ≥ 4 log_10_ reduction steps for yeast relative to the initial microbial counts, to achieve a reduction rate deemed “high”. Statistically significant reductions are marked at the first time point that significance was reached relative to the initial microbial counts, as indicated by different symbols for each tested antimicrobial (‘*’ ≙ CPC, ‘°’ ≙ MST, ‘§’ ≙ CHX, ‘#’ ≙ PHMB, ‘&’ ≙ PVP-I, ‘$’ ≙ OCT/PE). Degree of significance is indicated with the number of symbols (one–*p* < 0.05; two–*p* < 0.01; three–*p* <0.001, four–*p* < 0.0001).

OCT/PE and PVP-I achieved significant and full reduction of all tested microorganisms within 0.5 min of exposure (*p* < 0.01). The increased protein challenges of 0.3% or 3% albumin did not exert a mitigating effect on their antimicrobial efficacy (Figures 1–5).

#### 3.1.2. PHMB

PHMB proved to be less effective in the conducted experiments. After 0.5 min of exposure with the 0.3% protein challenge, PHMB achieved significantly lower reduction rates compared to the other tested antimicrobials, such as OCT/PE or PVP-I (*p* < 0.01). A significant and strong antimicrobial efficacy (>5 log_10_ steps; *p* < 0.05) was achieved against all investigated microorganisms; but, for full reduction, extended exposure times were necessary: 3 min for *S. aureus* (Figure 1) and *P. aeruginosa* (Figure 3) and 5 min for *E. faecium* (Figure 4) and *C. albicans* (Figure 5). Only against *E. coli* (Figure 3) did PHMB not achieve complete reduction within the investigated exposure time (maximum reduction: 7.38 ± 0.81).

**Figure 6.**
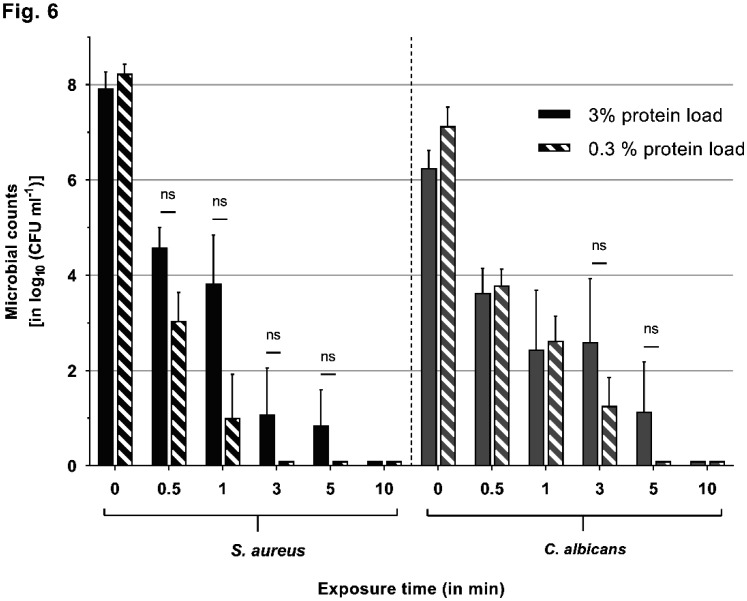
Side-by-side comparison of differences in reduction rates under the 3% (high) or 0.3% (low) protein load (exemplary for PHMB). Microbial counts are expressed as log_10_ CFU mL^−1^ over time. Reduction rates of PHMB, as an exemplary antimicrobial, are displayed against *S. aureus* (left side) and *C. albicans* (right side). Results are displayed in a side-by-side comparison, where the filled bars depict the results for the increased (3% albumin) protein load, and the striped bars show the results for the lower (0.3% albumin) protein load. Differences in efficacy can be observed between 0.5 and 5 min of exposure, with decreased reduction rates for the higher protein load of up to 2.8 log_10_ steps in magnitude (against *S. aureus* after 1 min). However, the results did not prove to be statistically significant in the performed experiments (as indicated by ‘ns').

Under the increased protein load (3% albumin), PHMB showed comparable overall results, achieving complete eradication for all tested microorganisms, except for *E. coli* (Figure 2). However, longer exposure times were necessary with the increased protein challenge, especially to reduce *S. aureus* and *C. albicans* (10 min for both), and reduction rates were lower at tested time points compared to the 0.3% protein challenge, although they were not statistically significant (Figure 6). Against *P. aeruginosa* and *E. faecium*, PHMB managed complete reduction within 3 min (Figures 3 and 4).

#### 3.1.3. CHX

CHX challenged with 0.3% albumin achieved complete reduction of *E. coli* (within 0.5 min; *p* = 0.0009, Figure 2), *P. aeruginosa* (within 1 min; *p* = 0.0148, Figure 3) and *E. faecium* (within 5 min; *p* = 0.0017, Figure 4) after varying exposure times. Against *S. aureus* and *C. albicans*, CHX did not manage complete reduction under the 0.3% protein load within the tested exposure time, but it achieved significant and required reduction rates (>5 log_10_ steps within 1 min; *p* < 0.05, Figures 1 and 5). The standard criterion was not met for *E. faecium* (Supplementary table 1 and Figure 4).

Under the 3% protein load, CHX achieved comparable results, demonstrating no significant difference in efficacy between the 0.3% and 3% challenge for most pathogens. Only for *S. aureus* was an extended exposure time needed to achieve the required cut-off (3 min; 5.99 ± 1.99, Figure 1).

#### 3.1.4. CPC

Regardless of the increased protein load, CPC achieved strong antimicrobial efficacy within 0.5 min of exposure, as indicated by the standard, against all tested microorganisms. Complete reduction was achieved within 0.5 min against *S. aureus*, *E. coli* and *E. faecium* (*p* < 0.01; Figures 1, 2 and 4). For *C. albicans* under the 0.3% challenge (Figure 5) and *P. aeruginosa* under the 3% challenge (Figure 3), 1 min of exposure was needed for complete reduction.

#### 3.1.5. MST

Against *E. coli* and *E. faecium*, MST managed complete reduction within 0.5 min under the lower (0.3%) and higher (3%) protein loads (Figures 2 and 4). Generally, MST met the criteria set by the standards (>5 log_10_ steps within 1 min) for all tested microorganisms.

Challenged with 0.3% albumin, MST managed complete reduction of *S. aureus* and *C. albicans* within 3 min (Figure 1 and 5; *p* < 0.01), while, for *P. aeruginosa*, 5 min of exposure was needed (Figure 3; *p* = 0.0068).

Under the higher protein load (3%), the results were similar to those for the 0.3% challenge, with MST achieving complete reduction of *S. aureus* and *C. albicans* within 3 min (Figures 1 and 5; *p* < 0.01). For *P. aeruginosa*, only 1 min of exposure was needed under the 3% albumin challenge (Figure 3; *p* = 0.0029).

## 4. Discussion

Due to the globally rising challenge of antibiotic resistance and tolerance development in microorganisms [12], advancements to face such challenges and investigations into new local antimicrobial agents are indispensable. Based on previously published “proof-of-concept” data on the basic efficacy of CPC and MST as alternative local antimicrobials and their biocompatibility profile to human regenerative skin and wound cells (keratinocytes and fibroblasts) [15], this study extended the previous work to evaluate CPC and MST by using a side-by-side comparison to establish antiseptics. To especially account for the challenging, high-protein environment of wounds, bovine albumin was used in the experimental setup as a challenge substance to evaluate the protein interference of the tested agents, which is capable of reducing an antimicrobial's efficacy [16],[17].

As expected, the established agents OCT/PE, PVP-I, CHX and PHMB managed the broad range of tested microorganisms well and achieved high antimicrobial efficacies, as reported in several previous studies [11],[20],[21]. All investigated agents, except PHMB and CHX in a few cases, achieved the required reduction of >5 log_10_ phases within 1 min of exposure, as demanded by the international standard, and managed complete microbial reduction within the investigated time-course. PHMB and CHX demonstrated an overall strong antimicrobial efficacy as well; however, PHMB failed to completely eradicate *E. coli* over this study's time-course, although it achieved a high reduction rate (Figure 2). CHX only demonstrated weaknesses against *S. aureus* and *C. albicans* (Figures 1 and 5). Of the established antimicrobials, OCT/PE and PVP-I proved to be most effective, followed by PHMB and CHX.

The alternative agents investigated, especially CPC, demonstrated an antimicrobial efficacy comparable to the most effective established antiseptics OCT/PE and PVP-I in the tested concentration and exposure times (Figures 1–5). Only in one case (against *P. aeruginosa* under the 3% protein load) did CPC need to be insignificantly longer to achieve complete reduction (1 min instead of 0.5 min). In all other experiments, CPC performed as well as OCT/PE and PVP-I, regardless of the administered protein load, proving that CPC does not suffer from protein interference. Especially, under the high protein challenge (3%) within clinically realistic and relevant short exposure times of 1–3 min, CPC demonstrated significantly higher and faster reduction rates than PHMB and CHX (Figure 1a–e; *p* < 0.05). MST, overall, showed a slightly lower efficacy and partly longer necessary exposure times than OCT/PE, PVP-I and CPC; however, they only showed statistical significance against *S. aureus* (*p* < 0.01) and *P. aeruginosa* (*p* < 0.01) within the first 0.5 min. Compared to PHMB and CHX, MST proved to be significantly more effective against most microorganisms (*p* < 0.05).

As described by various studies, several antiseptics show a significant loss of efficacy in high-protein environments. The local wound microenvironment contains about 3 to 5% total protein in acute and chronic wounds [22],[23]. When assessing antimicrobial agents, this influential factor needs to be considered to approximate their efficacy in the in-vivo setting. Kapalschinski et al. demonstrated that the addition of 0.3% albumin in in-vitro settings already significantly reduces the antiseptic potency of PHMB, OCT/PE and PVP-I, depending on the antiseptic's concentration, and that, with a rising protein concentration, the antimicrobial efficacy is further diminished [16],[17]. These results are in line with our previous investigations of antiseptic wound dressings, demonstrating a significant dose-dependent loss of efficacy for some silver- and PHMB-containing foam dressings challenged with human acute wound fluid (median protein content of 3.9%) [18], as well as the results in this study; especially, the biguanides PHMB and CHX, to some extent, demonstrate differences in performance under the simple 0.3% and 3% albumin challenges (e.g., PHMB against *S. aureus* (Figure 6)). The fact that this only occurred for PHMB and CHX without statistical significance presumably arises from the differences in evaluated concentrations. The reported significant results of Kapalschinski et al. became especially apparent in diluted concentrations of the tested antiseptics (e.g., 0.005% PHMB), while the concentrations of other publications and commercially available products is usually higher (0.02%, 0.04% or higher) and less affected by the investigated lower amounts of simple and most singular (albumin) protein loads. Nevertheless, 0.02% PHMB yielded comparable decreases in reduction rates (~1 log_10_ difference between 0.3% and 3% albumin) against *S. aureus* in both studies. The tested concentrations of OCT/PE and PVP-I showed no reduction in efficacy under the high protein challenge, which is also in line with previously reported results [16]. The alternatives of interest, CPC and MST, demonstrated somewhat varying results: while the efficacy of CPC remained unaltered under the higher protein load, MST showed some decrease in efficacy; however, it was only against *S. aureus* within the first 0.5 min of exposure (~3 log_10_ reductions; Figure 1). Such minor differences on this level might be negligible. Therefore, in this study, no protein interference could be observed for CPC, OCT/PE and PVP-I, while, for MST, a low protein interference, and for CHX and PHMB, a comparably higher protein interference, became apparent in the tested concentrations. Generally, the reported results indicate a certain extent of protein interference in a complex high-protein microenvironment of the wound that is dependent on the concentration of the used antimicrobial, the amount of challenging total protein and the class of antimicrobial. For biguanides such as CHX and PHMB, a high protein load seems to be more relevant than for other antimicrobials.

However, especially in the case of PHMB, the results have to be interpreted with regard to the purposely used lower concentration of PHMB (0.02%). The general concentrations used for PHMB in chronic wound management are 0.02%, 0.04% or even 0.1% in acute infections [24], expectedly yielding faster and higher reduction rates with rising concentration. However, for higher concentrations, accordingly higher cytotoxicity has been reported [10],[25]. Therefore, in this study, the lower, yet still highly effective, concentration of 0.02% was chosen in order to compare concentrations of CPC, MST and PHMB with similar cytotoxicity levels. Another reason for the lower efficacy of PHMB in this study might be the previously reported longer onset of the full efficacy of PHMB, making longer exposure times necessary for complete reduction in the case of PHMB usage. Therefore, the results are somewhat limited to a comparison in light of the specific antimicrobials and their concentrations used. Higher concentrations yield higher potential toxic impact on human skin and wound cells since antimicrobial efficacy and cytotoxicity is inseparable. Since the intention of the presented work was to evaluate the comparability and non-inferiority of the alternative agents CPC and MST in vitro, especially with regard to a potential interference with protein, the concentrations were selected to achieve a high reduction under unchallenged conditions, while at the same time, demonstrating an acceptable and similar biocompatibility to established antiseptics. This is particularly true for PHMB, which is currently the antimicrobial agent of choice for chronic wounds, making it the “benchmark substance” [24]. At the same time, highly effective agents for fighting local infections, such as OCT/PE, demonstrated higher cytotoxicity in previous studies [10], resulting in the need to carefully balance their application, especially in chronic wounds, to prevent healing impairment. Therefore, newly introduced alternative agents should ideally match the antiseptic efficacy of OCT/PE while exerting lower toxicity to human skin and wound cells.

In light of the presented results, the antimicrobial efficacy of CPC and MST, unimpeded by a high protein load (3%), proved to be equal (or even superior) to established antimicrobials in a dose-dependent manner when tested against a broad range of microorganisms encountered in wound management. Regarding antimicrobial efficacy, CPC demonstrated the same efficacy as the highly potential antiseptics OCT/PE and PVP-I with no “protein error”. MST proved to be only slightly less effective, yet it was still equally or more effective than the biguanides PHMB and CHX. In the context of biocompatibility with human wound cells, the alternative agents demonstrated promising toxicity profiles in our group's earlier published in-vitro evaluations [15]. For OCT/PE and PVP-I, severe cytotoxic effects on human keratinocytes and fibroblasts in vitro have been reported [21], with the reports even demonstrating dilutions of commercially available products as low as 12.5% (OCT/PE) and 7.5% (PVP-I) to reduce cell viability and proliferation of fibroblasts and keratinocytes to 0% [26]. MST (0.05%), in comparison, demonstrated a relevantly higher cell vitality of about 30% for keratinocytes and fibroblasts in a previous study [15], still demonstrating a comparable degree of toxicity; however, it was far more biocompatible than OCT/PE or PVP-I, with comparable antimicrobial efficacy. CPC proved to be even less toxic, showing only a 50% reduced cell vitality within 1 h of exposure [15], as well as a toxicity level for human skin cells that was considerably lower than that for OCT/PE or PVP-I, while achieving the same antimicrobial efficacy without protein interference. Compared to the more tolerable PHMB, CPC also demonstrated a comparable cytotoxicity, as well as a comparable antimicrobial efficacy, assuming higher concentrations of PHMB (e.g., 0.04%) used.

Nonetheless, it needs to be emphasized that these are in vitro results, laying the foundation for more detailed evaluations of the alternative agents, especially those using in-vivo and clinical settings.

## 5. Conclusions

The presented work demonstrated CPC and MST to be antimicrobial agents of comparable efficacy as well-established products. In comparison to PHMB and CHX, they even surpass their antimicrobial efficacy. Thereby, no relevant interference with the high protein challenge in terms of a “protein error” could be observed. This evaluation is intended to work toward extending the available array of highly efficient antiseptics and antimicrobials in wound care. Overall, CPC and MST feature a high potential as new, alternative anti-infectives in wound care based on their evaluated efficacy profiles. Especially, CPC should be further pursued in more complex in-vitro and comprehensive in-vivo studies, as well as in biocompatibility analyses, as a potential additional antimicrobial agent in wound management.

## References

[1] Hachenberg T, Senturk M, Jannasch O (2010). Postoperative wound infections. Pathophysiology, risk factors and preventive concepts. Anaesthesist.

[2] Malone M, Bjarnsholt T, McBain AJ (2017). The prevalence of biofilms in chronic wounds: a systematic review and meta-analysis of published data. J Wound Care.

[3] Werdin F, Tennenhaus M, Schaller HE (2009). Evidence-based management strategies for treatment of chronic wounds. Eplasty.

[4] Zarb P, Coignard B, Griskeviciene J (2012). The European Centre for Disease Prevention and Control (ECDC) pilot point prevalence survey of healthcare-associated infections and antimicrobial use. Euro Surveill.

[5] ESPAUR (2017). English Surveillance Programme for Antimicrobial Utilisation and Resistance (ESPAUR)-Report 2017.

[6] NRZ (2016). German national point-prevalence survey on nosocomial infections and antibiotic utilisation 2016.

[7] Lachapelle JM (2014). A comparison of the irritant and allergenic properties of antiseptics. Eur J Dermatol.

[8] Norman G, Dumville JC, Moore ZE (2016). Antibiotics and antiseptics for pressure ulcers. Cochrane Database Syst Rev.

[9] Wu L, Norman G, Dumville JC (2015). Dressings for treating foot ulcers in people with diabetes: an overview of systematic reviews. Cochrane Database Syst Rev.

[10] Hirsch T, Koerber A, Jacobsen F (2010). Evaluation of toxic side effects of clinically used skin antiseptics in vitro. J Surg Res.

[11] Hirsch T, Seipp HM, Jacobsen F (2010). Antiseptics in surgery. Eplasty.

[12] Ventola CL (2015). The antibiotic resistance crisis: part 1: causes and threats. P T.

[13] Bowler PG (2018). Antibiotic resistance and biofilm tolerance: a combined threat in the treatment of chronic infections. J Wound Care.

[14] Percival SL, Salisbury AM, Chen R (2019). Silver, biofilms and wounds: resistance revisited. Crit Rev Microbiol.

[15] Fromm-Dornieden C, Rembe JD, Schafer N (2015). Cetylpyridinium chloride and miramistin as antiseptic substances in chronic wound management - prospects and limitations. J Med Microbiol.

[16] Kapalschinski N, Seipp HM, Kuckelhaus M (2017). Albumin reduces the antibacterial efficacy of wound antiseptics against Staphylococcus aureus. J Wound Care.

[17] Kapalschinski N, Seipp HM, Onderdonk AB (2013). Albumin reduces the antibacterial activity of polyhexanide-biguanide-based antiseptics against Staphylococcus aureus and MRSA. Burns.

[18] Rembe JD, Fromm-Dornieden C, Bohm J (2018). Influence of human acute wound fluid on the antibacterial efficacy of different antiseptic polyurethane foam dressings: An in vitro analysis. Wound Repair Regen.

[19] DIN (2013). EN 13727:2012+A1:2013-Chemical disinfectants and antiseptics–quantitative suspension test for the evaluation of bactericidal activity in the medical area–test method and requirements (phase 2, step 1); German version.

[20] Hirsch T, Limoochi-Deli S, Lahmer A (2011). Antimicrobial activity of clinically used antiseptics and wound irrigating agents in combination with wound dressings. Plast Reconstr Surg.

[21] Muller G, Kramer A (2008). Biocompatibility index of antiseptic agents by parallel assessment of antimicrobial activity and cellular cytotoxicity. J Antimicrob Chemother.

[22] James TJ, Hughes MA, Cherry GW (2000). Simple biochemical markers to assess chronic wounds. Wound Repair Regen.

[23] Thamm OC, Koenen P, Bader N (2015). Acute and chronic wound fluids influence keratinocyte function differently. Int Wound J.

[24] Kramer A, Dissemond J, Kim S (2018). Consensus on Wound Antisepsis: Update 2018. Skin Pharmacol Physiol.

[25] Rembe JD, Fromm-Dornieden C, Schafer N (2016). Comparing two polymeric biguanides: chemical distinction, antiseptic efficacy and cytotoxicity of polyaminopropyl biguanide and polyhexamethylene biguanide. J Med Microbiol.

[26] Hirsch T, Jacobsen F, Rittig A (2009). A comparative in vitro study of cell toxicity of clinically used antiseptics. Hautarzt.

