# Supplementary material for: Antimicrobials cetylpyridinium-chloride and miramistin demonstrate non-inferiority and no “protein-error” compared to established wound care antiseptics in vitro
Source: AIMS Microbiol. 2022 Oct 24;8(4):372–87. doi: 10.3934/microbiol.2022026 (PMC9834083; doi:10.3934/microbiol.2022026)
Supplement: Supplementary file 1 [file microbiol-08-04-026-s001.pdf]

AIMS Microbiology

AIMS Microbiology, 8(4): 372–387.
DOI: 10.3934/microbiol.2022026
Received: 26 July 2022
Revised: 02 October 2022
Accepted: 17 October 2022
Published: 24 October 2022

http://www.aimspress.com/journal/microbiology

Research article

Antimicrobials cetylpyridinium-chloride and miramistin demonstrate non-inferiority and no "protein-error "compared to established wound care antiseptics *in-vitro*

Julian-Dario Rembe[1,*], Vivian-Denise Thompson[2] and Ewa Klara Stuermer[3]

[1] Department of Vascular and Endovascular Surgery, Medical Faculty and University Hospital Duesseldorf, Heinrich-Heine-University, Duesseldorf, North-Rhine Westphalia, Germany
[2] Chair for Translational Wound Research, Centre for Biomedical Education and Research, Witten/Herdecke University, Witten, North-Rhine Westphalia, Germany
[3] Department of Vascular Medicine, University Heart Center, University Medical Center Hamburg-Eppendorf (UKE), Hamburg, Germany

* **Correspondence:** Email: julian-dario.rembe@med.uni-duesseldorf.de.

Supplementary Table 1. Overview microbial reduction rates. Reduction rates (in Δlog_{10} CFU ml^{-1}) for all tested antimicrobials calculated in relation to initial microbial counts against tested microorganisms (*S. aureus*, *P. aeruginosa*, *E. coli*, *E. faecium*, *C. albicans*) over a total 10 min of exposure with either 0.3% or 3% protein load. Values are expressed as means ± SEM. Grey-scaling indicates levels of antimicrobial reduction: no color indicates < 5 Δlog_{10} CFU ml^{-1}, light grey indicates minimum required reduction (≥ 5 Δlog_{10} CFU ml^{-1} for bacteria as specified by DIN EN 13727 or ≥ 4 Δlog_{10} CFU ml^{-1} for yeast as specified in DIN EN 13624) and dark grey indicates complete reduction of initial microbial counts.

	Octenidine/phenoxyethanol (OCT/PE)									
	0.3 % Albumin challenge					3 % Albumin challenge				
time (min)	0.5	1	3	5	10	0.5	1	3	5	10
S. aureus	8.02±0.29	8.02±0.29	8.02±0.29	8.02±0.29	8.02±0.29	7.55±0.36	7.55±0.36	7.55±0.36	7.55±0.36	7.55±0.36
P. aeruginosa	8.70±0.65	8.70±0.65	8.70±0.65	8.70±0.65	8.70±0.65	8.61±0.16	8.61±0.16	8.61±0.16	8.61±0.16	8.61±0.16
E. coli	8.13±0.16	8.13±0.16	8.13±0.16	8.13±0.16	8.13±0.16	8.55±0.36	8.55±0.36	8.55±0.36	8.55±0.36	8.55±0.36
E. faecium	7.42±0.19	7.42±0.19	7.42±0.19	7.42±0.19	7.42±0.19	7.67±0.21	7.67±0.21	7.67±0.21	7.67±0.21	7.67±0.21
C. albicans	7.13±0.40	7.13±0.40	7.13±0.40	7.13±0.40	7.13±0.40	6.74±0.26	6.74±0.26	6.74±0.26	6.74±0.26	6.74±0.26
	Povidone-iodine (PVP-I)									
	0.3 % Albumin challenge					3 % Albumin challenge				
time (min)	0.5	1	3	5	10	0.5	1	3	5	10
S. aureus	8.02±0.29	8.02±0.29	8.02±0.29	8.02±0.29	8.02±0.29	7.93±0.34	7.93±0.34	7.93±0.34	7.93±0.34	7.93±0.34
P. aeruginosa	8.69±0.65	8.69±0.65	8.69±0.65	8.69±0.65	8.69±0.65	8.61±0.16	8.61±0.16	8.61±0.16	8.61±0.16	8.61±0.16
E. coli	8.18±0.22	8.18±0.22	8.18±0.22	8.18±0.22	8.18±0.22	8.51±0.34	8.51±0.34	8.51±0.34	8.51±0.34	8.51±0.34
E. faecium	7.42±0.19	7.42±0.19	7.42±0.19	7.42±0.19	7.42±0.19	7.67±0.21	7.67±0.21	7.67±0.21	7.67±0.21	7.67±0.21
C. albicans	7.13±0.40	7.13±0.40	7.13±0.40	7.13±0.40	7.13±0.40	6.74±0.26	6.74±0.26	6.74±0.26	6.74±0.26	6.74±0.26
	Polyhexanide (PHMB)									
	0.3 % Albumin challenge					3 % Albumin challenge				
time (min)	0.5	1	3	5	10	0.5	1	3	5	10
S. aureus	5.19±0.79	7.22±1.09	8.23±0.20	8.23±0.20	8.23±0.20	3.35±0.73	4.10±1.32	6.92±1.35	7.08±1.09	7.93±0.34
P. aeruginosa	6.89±1.31	7.28±0.95	8.41±0.44	8.41±0.44	8.41±0.44	5.90±1.49	6.86±0.99	8.61±0.16	8.61±0.16	8.61±0.16
E. coli	5.26±1.43	5.52±1.35	6.81±1.28	6.96±1.13	7.31±0.78	5.67±1.55	6.12±1.32	6.60±1.20	6.92±1.06	7.75±1.00
E. faecium	4.88±1.48	5.20±1.33	6.72±0.73	7.42±0.19	7.42±0.19	4.55±0.07	5.55±0.07	7.67±0.21	7.67±0.21	7.67±0.21
C. albicans	3.35±0.27	4.50±0.21	5.87±0.62	7.13±0.40	7.13±0.40	2.62±0.19	3.82±1.16	3.65±1.24	5.11±0.74	6.25±0.37
	Chlorhexidine (CHX)									
	0.3 % Albumin challenge					3 % Albumin challenge				
time (min)	0.5	1	3	5	10	0.5	1	3	5	10
S. aureus	4.65±0.56	5.17±0.39	6.16±0.72	7.06±0.62	7.22±0.45	3.31±0.08	4.90±0.04	5.95±1.12	6.16±1.02	7.33±0.45
P. aeruginosa	6.22±0.35	8.70±0.65	8.70±0.65	8.70±0.65	8.70±0.65	5.73±0.58	8.99±0.31	8.99±0.31	8.99±0.31	8.99±0.31
E. coli	8.13±0.16	8.13±0.16	8.13±0.16	8.13±0.16	8.13±0.16	8.45±0.31	8.45±0.31	8.45±0.31	8.45±0.31	8.45±0.31
E. faecium	3.30±0.33	4.44±0.65	6.58±0.86	7.42±0.19	7.42±0.19	3.66±0.27	4.33±0.10	6.85±0.82	7.69±0.24	7.69±0.24
C. albicans	3.03±0.43	4.40±1.00	5.91±0.79	6.08±0.63	6.53±0.24	4.64±0.75	5.44±0.36	6.74±0.26	6.74±0.26	6.74±0.26

Continued on next page

	Cetylpyridinium-chloride (CPC)									
	0.3 % Albumin challenge					3 % Albumin challenge				
time (min)	0.5	1	3	5	10	0.5	1	3	5	10
S. aureus	8.02±0.29	8.02±0.29	8.02±0.29	8.02±0.29	8.02±0.29	7.87±0.31	7.87±0.31	7.87±0.31	7.87±0.31	7.87±0.31
P. aeruginosa	8.41±0.44	8.41±0.44	8.41±0.44	8.41±0.44	8.41±0.44	8.16±0.57	8.71±0.13	8.71±0.13	8.71±0.13	8.71±0.13
E. coli	8.26±0.03	8.26±0.03	8.26±0.03	8.26±0.03	8.26±0.03	8.62±0.23	8.62±0.23	8.62±0.23	8.62±0.23	8.62±0.23
E. faecium	7.42±0.19	7.42±0.19	7.42±0.19	7.42±0.19	7.42±0.19	7.38±0.07	7.38±0.07	7.38±0.07	7.38±0.07	7.38±0.07
C. albicans	6.37±0.36	7.13±0.40	7.13±0.40	7.13±0.40	7.13±0.40	6.31±0.43	6.31±0.43	6.31±0.43	6.31±0.43	6.31±0.43
	Miramistin (MST)									
	0.3 % Albumin challenge					3 % Albumin challenge				
time (min)	0.5	1	3	5	10	0.5	1	3	5	10
S. aureus	6.57±1.55	7.53±0.61	8.13±0.21	8.13±0.21	8.13±0.21	4.43±0.96	6.49±0.79	7.75±0.33	7.75±0.33	7.75±0.33
P. aeruginosa	6.75±1.05	6.93±0.97	6.79±1.30	8.41±0.44	8.41±0.44	6.95±0.55	8.99±0.31	8.99±0.31	8.99±0.31	8.99±0.31
E. coli	8.26±0.03	8.26±0.03	8.26±0.03	8.26±0.03	8.26±0.03	8.57±0.27	8.57±0.27	8.57±0.27	8.57±0.27	8.57±0.27
E. faecium	7.42±0.19	7.42±0.19	7.42±0.19	7.42±0.19	7.42±0.19	7.38±0.12	7.38±0.12	7.38±0.12	7.38±0.12	7.38±0.12
C. albicans	5.30±1.39	5.86±0.84	7.13±0.40	7.13±0.40	7.13±0.40	4.95±0.89	5.43±0.43	6.31±0.43	6.31±0.43	6.31±0.43

AIMS Press

© 2022 the Author(s), licensee AIMS Press. This is an open access article distributed under the terms of the Creative Commons Attribution License (http://creativecommons.org/licenses/by/4.0)
